# Elderly ATL patients in ageing society of Japan

**DOI:** 10.1186/1742-4690-8-S1-A32

**Published:** 2011-06-06

**Authors:** Shigeki Takemoto, Ratiorn Pornkuna, Yusuke Higuchi, Takahiro Matsui, Toshio Kawakita, Miki Nakamura, Yoshiko Inoue, Tatsunori Sakai, Naoko Harada, Shoichi Nagakura, Michihiro Hidaka, Tetsuyuki Kiyokawa, Fumio Kawano

**Affiliations:** 1 Laboratory of Special Diseases, Institute for Clinical Research, National Hospital Organization Kumamoto Medical Center, Kumamoto, Japan; 2 Department of Hematology, National Hospital Organization Kumamoto Medical Center, Kumamoto, Japan

## Background

The increase in the number of elderly people results in the exponential increase of cancer incidence, though we are wondering how that of ATL change.

## Materials and method

We assessed the therapy and outcome of newly diagnosed or referred 66 ATL patients from 2006 to 2009. Serum of patient was preserved in freezer to detect soluble proteins, sCD30 and sIL-2R, using ELISA.

## Results

More than 70% of newly diagnosed ATL patients were elderly persons aged 65 or older. Twenty-three of 66 patients at age from 41 to 61-year-old underwent allogeneic hematopoietic stem cell transplantation (allo-HSCT) in our hospital. On the other hand, 38 patients were not suitable for allo-HSCT therapy. Their ages were from 51 to 89-year-old and elderly aged 65-year-old and over occupied 73.3% of them (22 of 30, who we confirmed their outcome). Seven patients of them died before treatment or during the primary therapy. Then, we focused patients cause acute transformation from chronic type to figure out the timing of primary intervention and the efficiency of the therapy. Elevating sCD30 was observed earlier than other markers (sIL-2R, LDH) due to acute transformation and reduced by the treatment regimen changed even during treatment of relapse (recurrence).

## Discussion

Retrospective monitoring of soluble protein in serum was found to be effective to confirm the progression of disease as well as the remission state following remission induction therapy and maintenance therapy (Nishioka C, Takemoto S, et al.). And sCD30 elevated earlier than the other markers indicating that it is useful to predict the onset of acute crisis in ATL and detect the timing of the chemotherapy beginning. Successful intervention at the best timing and change of therapy in aggressive phase and indolent phase, respectively, resulted in disappearance of ATL cells and accomplished complete remission as long as we can.

## Conclusions

Changes in ATL patients by the advent of aged society indicate early therapeutic intervention and selection of appropriate treatment is required. If patients relapse after chemotherapy, a new therapy with an anti-CCR4 antibody, which is under development, may become an option, because our elderly patient achieved remission state by this therapy in the clinical study.
